# Transition from Transrectal to Transperineal MRI-Fusion Prostate Biopsy Does Not Comprise Detection Rates of Clinically Significant Prostate Cancer at a Tertiary Care Center

**DOI:** 10.3390/diagnostics14111184

**Published:** 2024-06-05

**Authors:** Benedikt Hoeh, Mike Wenzel, Clara Humke, Cristina Cano Garcia, Carolin Siech, Melissa Schneider, Carsten Lange, Miriam Traumann, Jens Köllermann, Felix Preisser, Felix K. H. Chun, Philipp Mandel

**Affiliations:** 1 Department of Urology, University Hospital Frankfurt, Goethe University Frankfurt am Main, 60590 Frankfurt am Main, Germany; 2 Cancer Prognostics and Health Outcomes Unit, Division of Urology, University of Montreal Health Center, Montreal, QC H2X 3E4, Canada; 3 Dr. Senckenberg Institute of Pathology, University Hospital Frankfurt, 60590 Frankfurt am Main, Germany; 4 Martini-Klinik Prostate Cancer Center, University Hospital Hamburg-Eppendorf, 20246 Hamburg, Germany

**Keywords:** transperineal, transrectal, targeted biopsy, systematic biopsy, prostate cancer

## Abstract

Background: A remarkable paradigm shift has emerged regarding the preferred prostate biopsy approach, favoring the transperineal (TP) over the transrectal (TR) approach due to the reduced risk of severe urinary tract infections. However, its impact on the detection of clinically significant prostate cancer (csPCa) remains unclear. Materials and methods: We relied on a prospectively maintained tertiary care database to identify patients who underwent either TP or TR prostate biopsy between 01/2014 and 12/2023. Of those, only patients with suspicious magnetic resonance imaging (MRI) PIRADS lesions (Likert-scale: 3,4,5) received MRI-targeted and systematic biopsies. Detection rates of csPCa (International Society of Urological Pathology [ISUP] ≥ 2) were compared between biopsy approach (TP vs. TR) according to index lesion. Subsequently, uni- and multivariable logistic regression models were applied to investigate the predictive status of the biopsy approach within each subcohort. Results: Of 2063 patients, 1118 (54%) underwent combined MRI-guided and systematic prostate biopsy and were included in the final cohort. Of those, 127 (11%) and 991 (89%) underwent TP vs. TR. CsPCa rates, regardless of differences in patients’ demographics and distribution of index PIRDAS lesions, did not differ statistically significantly and were 51 vs. 52%, respectively (*p* = 0.8). CsPCa detection rates for PIRDAS-3, PIRADS-4 and PIRADS-5 did not differ and were 24 vs. 23%, 48 vs. 51% and 72 vs. 76% for PIRADS-3, PIRADS-4 and PIRADS-5 subgroups for TP vs. TR, respectively (all *p* ≥ 0.9) Conclusions: The current results support the available data indicating that TP biopsy approach is comparable to transrectal biopsy approach regarding csPCa detection rates.

## 1. Introduction

Among all malignancies, prostate cancer is the second most commonly diagnosed malignancy worldwide and the most frequently diagnosed cancer in Europe [1,2]. In the diagnostic pathway for suspected prostate cancer, prostate biopsy has demonstrated itself inarguably as the fundamental cornerstone of diagnostic assessment to confirm disease presence and histological type [3]. The introduction and broad acceptance of (pre-biopsy) multiparametric magnetic imaging (MRI) has marked a new era regarding prostate cancer diagnostic workup, evidenced by several pivotal trials and meta-analyses comparing systematic and MRI-targeted prostate biopsy in regard to detection rates in the recent past [4,5,6,7]. Until recently, the transrectal approach was the standard method for prostate biopsy. However, due to uprising efforts to minimize side effects and (infectious) complications following transrectal prostate biopsy, a transition towards a transperineal prostate biopsy approach has been seen in the recent past. In 2023, the European Association of Urology (EAU), as the only guideline association, ‘strongly’ recommended transperineal biopsies as the preferred technique, primarily because of comparable detection rates, yet their lower risk of severe urinary tract infections in comparison to more conservative recommendations (Grade C level) ushered by the National Institute for Health and Care Excellence (NICE) in the United Kingdom or by the American Association of Urology (AUA) [2,8,9]. With emerging insights indicating a reduction in infectious complications favoring the transperineal approach over the transrectal approach [10], both trial-derived and real-world data comparing the detection rate of clinically significant prostate cancer between transrectal and transperineal biopsy is still inconclusive and partly controversial [10,11,12,13,14,15,16]. To address this void, the current study seeks to contribute to the ongoing debate by reporting detection rates comparing both prostate biopsy approaches in a real-world study population of patients undergoing MRI-guided prostate biopsy at a tertiary care center.

## 2. Material and Methods

### 2.1. Study Population

**Figure 1:**
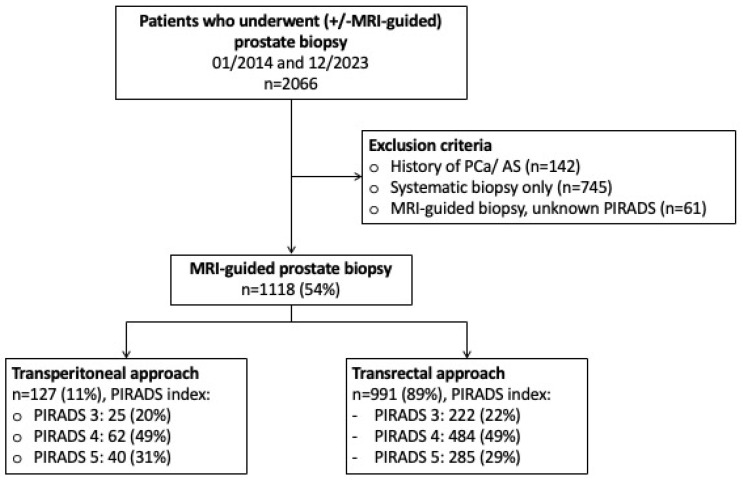
Flowchart depicting in- and exclusion criteria of the study population. Abbreviations: MRI = magnetic resonance imaging; PCa = prostate cancer; AS = active surveillance; PIRADS: Prostate Imaging Reporting and Data System.

Following approval of the institutional review boards of the University Cancer Centre Frankfurt and the Ethical Committee at the University Hospital Frankfurt (SUG-2-2018_A2023), patients who obtained a transrectal or transperineal MRI-guided prostate biopsy between 01/2014 and 12/2023 were retrospectively identified within our prospectively maintained database. As previously described patients with suspicious MRI findings (PIRADS 3, PIRADS 4, PIRADS 5) according to the Prostate Imaging Reporting & Data System [PIRADS Version 2.0, PIRADS Version 2.1] underwent MRI-guided biopsy of the lesion combined with a systematic prostate biopsy [17,18]. In the case of multiple lesions, MRI-guided biopsies covered all suspicious lesions separately according to standardized institutional protocol [19,20]. MRI-guided biopsy was performed with two high-end ultrasound machines (Transrectal: HiVison, Hitachi Medical Systems, Tokyo, Japan; transperineal: KOELIS Trinity System, Koelis, La Tronche, France). For MRI-guided biopsy, at least two cores were taken from each mpMRI lesion ≥ PIRADS 3. Between 01/2014 and 05/2023 (systematic and MRI-guided) prostate biopsies were performed via a transrectal approach (Hitachi Medical Systems), which was transitioned to a transperineal approach (Koelis) beginning in 06/2023. Among all patients who underwent prostate biopsy between 01/2014 and 12/2023 (*n* = 2063), patients with a previous history of prostate cancer (*n* = 142) were excluded. Subsequently, patients undergoing solely systematic prostate biopsy (*n* = 745) as well as patients with unknown PIRADS information (*n* = 61) were excluded, resulting in the final study cohort of 1118 patients obtaining a combined (MRI-guided and systematic) prostate biopsy in our tertiary care center (Figure 1).

### 2.2. Outcome Measurement

The outcome of interest was defined as the detection rate of clinically significant prostate cancer following prostate biopsy. Clinically significant prostate cancer was defined as Gleason score ≥ 3 + 4 or ISUP grade group ≥ 2 as previously published [2,21].

### 2.3. Statistical Analyses

The statistical analyses consisted of the following steps: First, the overall study population, irrespective of PIRADS index lesion, was tabulated according to the biopsy approach (transrectal vs. transperineal). Here, descriptive statistics included frequencies and proportions for categorical variables. Medians and interquartile ranges (IQR) were reported for continuously coded variables. The chi-square test examined the statistical significance of the differences in proportions while the Kruskal–Wallis test was used to examine differences in medians. Detection rates of clinically significant prostate cancer were calculated within the overall cohort. Subsequently, patients were stratified according to the index PIRADS lesions (Likert scale: PIRADS 3, PIRADS 4, PIRADS 5) and within each subgroup above mentioned tabulations were repeated. Finally, separate uni- and multivariable logistic regression models were fitted to test for a relation between the biopsy approach (transrectal vs. transperineal) and the detection of clinically significant prostate cancer. Adjustment variables consisted of age (continuously coded), prostate volume (continuously coded), PSA at biopsy (continuously coded), digital rectal examination (suspicious vs. non-suspicious), history of prior prostate biopsy (categorically coded), number of PIRADS lesions (continuously coded), number of lesion cores harbored (continuously coded). All tests were two-sided with a level of significance set a *p* < 0.05 and R-software environment for statistical computing and graphics (version 3.4.3) was used for all analyses.

## 3. Results

### 3.1. Descriptive Characteristics of the Study Population

Between 01/2014 and 12/2023, 1118 patients underwent combined MRI-guided and systematic prostate biopsy at our tertiary care center. Of those, 991 (89%) vs. 127 (11%) underwent transrectal vs. transperineal prostate biopsy, respectively (Table 1). Within the overall study cohort, the median age was 66 years (IQR: 60–72), the median PSA at biopsy was 7 ng/mL (IQR: 5–10) and the median prostate volume was 48 mL (IQR: 35–70) and did not differ between the patients undergoing transrectal vs. transperineal prostate biopsy. Moreover, no statistically significant differences were recorded in regard to digital rectal examination results nor to the distribution of PIRADS index lesions between the transrectal and transperineal approaches (all *p* > 0.05).

### 3.2. Detection Rates of Clinically Significant Prostate Cancer

Relying on the overall study cohort, the detection rate of clinically significant prostate cancer was 52% and did not differ between the transrectal and transperineal approaches (52 vs. 51%, *p* = 0.8). In subgroup analysis stratifying according to PIRADS index lesions (Tables S1–S3), detection rates between transrectal and transperineal approaches did not statistically significantly and were 23 vs. 24%, 51 vs. 48% and 76 vs. 72% for PIRADS 3, PIRADS 4 and PIRADS 5 subgroups, respectively (all *p* ≥ 0.9, Table 2). In separate uni- and multivariable logistic regression analyses, the transperineal approach (ref. transrectal approach) was not associated with lower detection rates of clinically significant prostate cancer throughout all subgroup, evidenced by a multivariable odds ratio of 0.64 (95%-CI [95% confidence interval]: 0.17–2.11; *p* = 0.49), 0.82 (95%-CI: 0.44–1.52; *p* = 0.52) and 0.63 (95%-CI: 0.24–1.71; *p* = 0.35) for PIRADS 3, PIRADS 4 and PIRADS 4 subgroups, respectively (Table 3).

## 4. Discussion

We hypothesized that a transition from transrectal to transperineal MRI-guided prostate biopsy would not compromise oncological outcomes, defined as the detection of clinically significant prostate cancer. To address this hypothesis, we relied on patients treated with MRI-guided transrectal vs. transperineal approach, of which the latter included all (consecutive) patients undergoing transperineal biopsy in the first six months following the introduction of the transperineal approach. We tested this hypothesis relying on 1118 patients and made some noteworthy findings.

The overall detection rate of clinically significant prostate cancer was 52% and did not statistically significantly differ between transperineal and transrectal (51% and 52%), respectively. The current results are in general agreement with previous reports comparing the transrectal and transperineal prostate biopsy approaches. For example, in the PReclude infection EVEnts with No prophylaxis Transperineal (PREVENT) randomized control trial, relying on 658 patients with prior MRI information, Hu et al. reported clinically significant prostate cancer detection rates of 53 vs. 51% for transperineal vs. transrectal approach, respectively [22]. It is of note, however, that the study population, as well as distribution of MRI findings and consequently MRI-targeted biopsies, differed between the study population by Hu et. and the current study [22]. Despite the findings in the current study as well as the study by Hu et al., Diamand et al. recently reported favorable clinically significant prostate cancer rates for transperineal compared to transrectal (51% vs. 45%) relying on a muti-centric study cohort of 3949 patients [14]. Even though the inclusion criteria—MRI-guided [PIRADS 3–5 lesion] with combined prostate biopsy—were comparable to the ones in the current study, differences in regard to the study populations and index PIRADS lesions distribution were prevalent [14].

Subgroup analyses focusing on lesion-specific clinically significant prostate cancer detection rates demonstrated comparable detection rates among PIRADS 3, PIRADS 4 and PIRADS 5 lesions, evidenced by clinically significant prostate cancer rates of 23 vs. 24%, 51 vs. 48% and 76 vs. 72% transrectal vs. transperineal, respectively (all *p* > 0.05). To account for potential confounding parameters as well as differences in patient and clinical characteristics between transrectal and transperineal patients, separate logistic regression models were fitted to investigate the predictive status of the biopsy approach in regard to clinically significant prostate cancer detection rate. Here, the transperineal approach was not associated with worse clinically significant prostate cancer detection rates, irrespectively of PIRADS index lesion in all separate logistic regression models (Table 3).

Taken together the current study underlines the feasibility of transitioning from transrectal to transperineal MRI-guided prostate biopsy without compromising detection rates of clinically significant prostate cancer.

Even though the current study carries important insights highlighting a feasible and oncological safe transition from transrectal to transperineal prostate biopsy, the current study is not devoid of limitations. First and foremost, the retrospective nature, limited sample size and the single-center tertiary care experience need to be taken into account when interpreting the results. Nevertheless, it should be highlighted that due to the single-center approach, solely one ‘institutional’ protocol was applied to all patients in contrast to multi-center study cohorts in which biases may arise due to differences in the institutional-specific protocols. Second, the current study solely focuses on oncological outcomes, namely detection rates of clinically significant prostate cancer. Even though secondary outcomes such as complication rates following TP or TR (with antibiotic prophylaxis) were not the focus of the current study, nevertheless represent important considerations [22,23]. Interestingly, in the PREVENT trial, post-biopsy infectious complication rates did not statistically significantly differ between TP vs. TR relying on a cohort of 658 patients [22]. Moreover, no statistically significant differences in infectious complication rates were recorded in the Prostate Biopsy: Efficacy and Complications (ProBE-PC) trial relying on 763 patients [23]. It is of note that differences in regard to the type and duration of antibiotic prophylaxis protocols as well as regional differences regarding antibiotic resistance should be taken into consideration when infectious complication rates represent the outcome of interest [24,25,26]. Additionally, patient-reported outcomes such as pain and discomfort as well as financial considerations were not included in the current study, yet represent other important considerations. It is of note that in the PREVENT trial, TP patients reported worse periprocedural pain compared to TR patients, yet the effect resolved within seven days post-biopsy [22]. Third, information regarding the exact number of cores and maximal lesion diameter as well as the exact location (anterior, posterior) of the index lesion is not available in the present study. However, data regarding its impact on the detection of clinically significant prostate cancer is inconclusive [10,27]. Fourth, among patients undergoing transrectal biopsy two different PIRADS versions (Version 2.0, Version 2.1) were used following its update in 2019 [17,18] whereas solely one PIRADS version (Version 2.1) was used for all transperineal patients resulting in a potential bias in regard to MRI reporting [18]. It is of note that in sensitivity analyses, including only transrectal patients with PIRADS Version 2.1 reporting information and comparing those to transperineal patients, results remained qualitatively unchanged. Finally, patients presented with either in-house or external MRI. As a consequence, differences in reporting quality, irrespective of PIRADS adherence and overall inter-reader agreement in interpretation, may be present in the current study [28].

## 5. Conclusions

The current results support the available data indicating that the transperineal biopsy approach is comparable to the transrectal biopsy approach regarding clinically significant prostate cancer detection rates in a tertiary care center and therefore transition from transrectal to transperineal MRI-guided prostate biopsy can safely be conducted from an oncological point of view.

## Figures and Tables

**Table 1:** Descriptive characteristics of patients undergoing MRI-guided prostate biopsy between 01/2014 and 12/2023; all values are median (IQR) and frequencies (%).

	*N*	Overall, *n* = 1118	Transrectal Biopsy, *n* = 991 (89%)	Transperineal Biopsy, *n* = 127 (11%)	*p*-Value
**Age at biopsy [years]** Median (IQR)	1118	66 (60, 72)	66 (60, 72)	67 (60, 72)	0.2
**Prostate-specific antigen [ng/mL]** Median (IQR)	1117	7 (5, 10)	7 (5, 10)	7 (5, 10)	0.2
**Prostate volume [mL]** Median (IQR)	1097	48 (35, 70)	50 (36, 70)	45 (35, 60)	0.087
**Total number of cores ** Median (IQR)	1113	15 (13, 17)	14 (13, 17)	17 (16, 19)	<0.001
**Number of cores: Systematic ** Median (IQR)	1113	12 (12, 12)	12 (12, 12)	12 (12, 12)	0.4
**Number of cores: PIRADS ** Median (IQR)	1114	3 (1, 5)	2 (1, 5)	5 (4, 7)	<0.001
**Digital rectal examination** *n* (%)	1118				0.057
Suspicous		250 (22%)	230 (23%)	20 (16%)	
Non-suspicous		868 (78%)	761 (77%)	107 (84%)	
**Number of prior** (**negative**) **biopsies** *n* (%)	1118				0.008
0		865 (77%)	753 (76%)	112 (88%)	
1		193 (17%)	181 (18%)	12 (9.4%)	
≥2		60 (5.4%)	57 (5.8%)	3 (2.4%)	
**Index PIRADS lesion** *n* (%)	1118				0.7
3		247 (22%)	222 (22%)	25 (20%)	
4		546 (49%)	484 (49%)	62 (49%)	
5		325 (29%)	285 (29%)	40 (31%)	
**Number of PIRADS lesions** *n* (%)	1118				0.075
1		800 (72%)	718 (72%)	82 (65%)	
≥2		318 (28%)	273 (28%)	45 (35%)	

Abbreviations: MRI = magnetic resonance imaging; PCa = Prostate cancer; AS = Active surveillance; PIRADS: Prostate Imaging Reporting and Data System; IQR = Interquartile range.

**Table 2:** Detection rates of clinically significant prostate cancer (defined as ISUP grade group ≥2 or Gleason score 3 + 4) according to MRI-guided biopsy approach stratified according to PIRADS index lesions.

	Overall Cohort				PIRADS 3				PIRADS 4				PIRADS 5			
	Overall	TR *n* = 991 (89%)	TP *n* = 127 (11%)	*p*-Value	Overall, *n* = 247	TR *n* = 222 (90%)	TP *n* = 25 (10%)	*p*-Value	Overall, *n* = 546	TR *n* = 484 (89%)	TP *n* = 62 (11%)	*p*-Value	Overall, *n* = 325	TR *n* = 285 (88%)	TP *n* = 40 (12%)	*p*-Value
**ISUP or** **GS at biopsy** *n* (%)				0.8				>0.9				0.9				>0.9
No PCa	358 (32%)	319 (32%)	39 (31%)		134 (54%)	121 (55%)	13 (52%)		174 (32%)	154 (32%)	20 (32%)		50 (15%)	44 (15%)	6 (15%)	
ISUP 1 or GS 3 + 3	179 (16%)	156 (16%)	23 (18%)		56 (23%)	50 (23%)	6 (24%)		95 (17%)	83 (17%)	12 (19%)		28 (8.6%)	23 (8.1%)	5 (12%)	
ISUP ≥ 2 or GS ≥ 3 + 4	581 (52%)	516 (52%)	65 (51%)		57 (23%)	51 (23%)	6 (24%)		277 (51%)	247 (51%)	30 (48%)		247 (76%)	218 (76%)	29 (72%)	

Abbreviations: PCa = prostate cancer; TR = transrectal prostate biopsy; TP = transperineal prostate biopsy; PIRADS = Prostate Imaging Reporting and Data System; GS = Gleason score.

**Table 3:** Separate univariable and multivariable logistic regression models investigating the predictor status of MRI-guided biopsy approach according to PIRADS index lesion.

		Univariable			Multivariable *		
		Odds Ratio	95%-CI	*p*-Value	Odds Ratio	95%-CI	*p*-Value
**PIRADS 3**	Biopsy approach						
	Transrectal	ref.			ref.		
	Transperineal	1.06	0.37–2.66	0.91	0.64	0.17–2.11	0.49
**PIRADS 4**	Biopsy approach						
	Transrectal	ref.			ref.		
	Transperineal	0.90	0.53–1.53	0.69	0.82	0.44–1.52	0.52
**PIRADS 5**	Biopsy approach						
	Transrectal	ref.			ref.		
	Transperineal	0.81	0.39–1.77	0.58	0.63	0.24–1.71	0.35

* Adjusted for age, prostate volume, PSA at biopsy, digital rectal examination, history of prior prostate biopsy, number of PIRADS lesions, number of biopsy cores harbored. Abbreviations: PIRADS = Prostate Imaging Reporting and Data System; PSA = prostate-specific antigen; 95%-CI = 95% confidence interval.

## Data Availability

All datasets generated for this study are included in the manuscript.

## Supplementary Materials

The following supporting information can be downloaded at: https://www.mdpi.com/article/10.3390/diagnostics14111184/s1, Supplementary Table S1. Descriptive characteristics of patients undergoing MRI-guided prostate biopsy with PIRADS 3 index lesion between 01/2014 and 12/2023; all values are median (IQR) and frequencies (%); Supplementary Table S2. Descriptive characteristics of patients undergoing MRI-guided prostate biopsy with PIRADS 4 index lesion between 01/2014 and 12/2023; all values are median (IQR) and frequencies (%); Supplementary Table S3. Descriptive characteristics of patients undergoing MRI-guided prostate biopsy with PIRADS 5 index lesion between 01/2014 and 12/2023; all values are median (IQR) and frequencies.
