# Supplementary material for: Transition from Transrectal to Transperineal MRI-Fusion Prostate Biopsy Does Not Comprise Detection Rates of Clinically Significant Prostate Cancer at a Tertiary Care Center
Source: Diagnostics (Basel). 2024 Jun 5;14(11):1184. doi: 10.3390/diagnostics14111184 (PMC11171881; doi:10.3390/diagnostics14111184)
Supplement: Supplementary file 1 [file diagnostics-14-01184-s001.zip › Supplementary Table 1.pdf]

Supplementary Table 1. Descriptive characteristics of patients undergoing MRI-guided prostate biopsy with PIRADS 3 index lesion between 01/2014 and 12/2023; all values are median (IQR) and frequencies (%);

	**N**	**Overall,** n= 247	**Transrectal biopsy,** n = 222 (90%)	**Transperineal biopsy,** n= 25 (10%)	**p-value**
**Age at biopsy [years]** Median (IQR)	247	63 (58, 69)	63 (58, 69)	62 (58, 65)	0.4
**Prostate volume [ml]** Median (IQR)	243	50 (35, 74)	51 (35, 75)	45 (35, 60)	0.5
**Prostate-specific antigen [ng/mL]** Median (IQR)	247	6.1 (4.5, 9.1)	6.2 (4.5, 9.0)	6.0 (4.5, 10.0)	0.6
**Total number of cores** Median (IQR)	246	15 (13, 16)	14 (13, 16)	16 (15, 17)	0.02
**Number of cores: Systematic** Median (IQR)	246	12 (12, 12)	12 (12, 12)	12 (12, 12)	0.8
**Number of cores: PIRADS-lesions** Median (IQR)	246	3 (1, 4)	2 (1, 4)	4 (3, 5)	0.001
**Digital rectal examination** n (%)	247				>0.9
Non-suspicous		232 (94%)	208 (94%)	24 (96%)	
Suspicous		15 (6.1%)	14 (6.3%)	1 (4.0%)	
**Number of prior (negative) biopsies** n (%)	247				0.032
0		184 (74%)	160 (72%)	24 (96%)	
1		46 (19%)	45 (20%)	1 (4.0%)	
≥2		17 (6.9%)	17 (7.7%)	0 (0%)	
**Number of PIRADS lesions** n (%)	247				>0.9
1		186 (75%)	167 (75%)	19 (76%)	
≥2		61 (25%)	55 (25%)	6 (24%)	

Abbreviations: MRI= magnetic resonance imaging; PCa= Prostate cancer; AS=Active surveillance; PIRADS: Prostate Imaging Reporting and Data System; IQR=Interquartile range;
