# Supplementary material for: Transition from Transrectal to Transperineal MRI-Fusion Prostate Biopsy Does Not Comprise Detection Rates of Clinically Significant Prostate Cancer at a Tertiary Care Center
Source: Diagnostics (Basel). 2024 Jun 5;14(11):1184. doi: 10.3390/diagnostics14111184 (PMC11171881; doi:10.3390/diagnostics14111184)
Supplement: Supplementary file 1 [file diagnostics-14-01184-s001.zip › Supplementary Table 2.pdf]

Supplementary Table 2. Descriptive characteristics of patients undergoing MRI-guided prostate biopsy with PIRADS 4 index lesion between 01/2014 and 12/2023; all values are median (IQR) and frequencies (%);

	N	**Overall,** n=546	**Transrectal biopsy,** n=484 (89%)	**Transperineal biopsy,** n=62 (11%)	**p-value**
**Age at biopsy [years]** Median (IQR)	546	66 (60, 72)	66 (60, 71)	67 (62, 73)	0.11
**Prostate volume [ml]** Median (IQR)	533	50 (35, 70)	50 (35, 70)	45 (35, 68)	0.2
**Prostate-specific antigen [ng/mL]** Median (IQR)	545	6.1 (4.5, 9.2)	6.1 (4.5, 9.2)	6.3 (4.8, 9.3)	0.5
**Total number of cores** Median (IQR)	544	15 (13, 17)	15 (13, 17)	17 (16, 20)	<0.001
**Number of cores: Systematic** Median (IQR)	544	12 (12, 12)	12 (12, 12)	12 (12, 12)	0.8
**Number of cores: PIRADS-lesions** Median (IQR)	544	3 (1, 5)	3 (1, 5)	5 (4, 8)	<0.001
**Digital rectal examination** n (%)	546				0.029
Non-suspicous		447 (82%)	390 (81%)	57 (92%)	
Suspicous		99 (18%)	94 (19%)	5 (8.1%)	
**Number of prior (negative) biopsies** n (%)	546				0.5
0		422 (77%)	370 (76%)	52 (84%)	
1		98 (18%)	90 (19%)	8 (13%)	
≥2		26 (4.8%)	24 (5.0%)	2 (3.2%)	
**Number of PIRADS lesions** n (%)	546				0.2
1		383 (70%)	344 (71%)	39 (63%)	
≥2		163 (30%)	140 (29%)	23 (37%)	

Abbreviations: MRI= magnetic resonance imaging; PCa= Prostate cancer; AS=Active surveillance; PIRADS: Prostate Imaging Reporting and Data System; IQR=Interquartile range;
