# Supplementary material for: Transition from Transrectal to Transperineal MRI-Fusion Prostate Biopsy Does Not Comprise Detection Rates of Clinically Significant Prostate Cancer at a Tertiary Care Center
Source: Diagnostics (Basel). 2024 Jun 5;14(11):1184. doi: 10.3390/diagnostics14111184 (PMC11171881; doi:10.3390/diagnostics14111184)
Supplement: Supplementary file 1 [file diagnostics-14-01184-s001.zip › Supplementary Table 3.pdf]

Supplementary Table 3. Descriptive characteristics of patients undergoing MRI-guided prostate biopsy with PIRADS 5 index lesion between 01/2014 and 12/2023; all values are median (IQR) and frequencies

	N	**Overall,** n=325	**Transrectal biopsy,** n=285 (88%)	**Transperineal biopsy,** n=40 (12%)	**p-value**[2]
**Age at biopsy [years]** Median (IQR)	325	68 (62, 74)	68 (62, 74)	68 (64, 74)	0.5
**Prostate volume [ml]** Median (IQR)	321	45 (37, 60)	46 (37, 60)	45 (37, 51)	0.5
**Prostate-specific antigen [ng/mL]** Median (IQR)	325	8 (5, 12)	8 (5, 12)	8 (6, 14)	0.4
**Total number of cores** Median (IQR)	323	14 (13, 17)	14 (13, 16.50)	17 (16, 19.25)	<0.001
**Number of cores: Systematic** Median (IQR)	323	12 (12, 12)	12 (12, 12)	12 (12, 12)	0.3
**Number of cores: PIRADS-lesions** Median (IQR)	324	3 (1, 5)	2 (1, 5)	5 (4.75, 7.25)	<0.001
**Digital rectal examination** n (%)	325				0.3
Non-suspicous		189 (58%)	163 (57%)	26 (65%)	
Suspicous		136 (42%)	122 (43%)	14 (35%)	
**Number of prior (negative) biopsies** n (%)	325				0.3
0		259 (80%)	223 (78%)	36 (90%)	
1		49 (15%)	46 (16%)	3 (7.5%)	
≥2		17 (5.2%)	16 (5.6%)	1 (2.5%)	
**Number of PIRADS lesions** n (%)	325				0.13
1		231 (71%)	207 (73%)	24 (60%)	
≥2		94 (29%)	78 (27%)	16 (40%)	

Abbreviations: MRI= magnetic resonance imaging; PCa= Prostate cancer; AS=Active surveillance; PIRADS: Prostate Imaging Reporting and Data System; IQR=Interquartile range;
